# Atlas to the Pathology of the Teeth

**Published:** 1869-04

**Authors:** 


### ATLAS TO THE PATHOLOGY OF THE TEETH.

In Four Parts. Arranged and explained by the late Prof. Dr. M. Heider, and Prof. Dr. C. Wedl.

Leipsic: London and New York.

Examination of the two numbers which have at present reached this country, shows the work to be one which has been much needed.

The illustrations which are drawings on stone from nature, are executed in the most beautiful manner. The contributions illustrating the formation of secondary dentine and its peculiarities are particularly interesting, and are by far the most valuable of any heretofore available to the pathologist or Dental student.

The microscopic appearance of the Dental pulp during chronic or local inflammation, as well as the resultant condition of fatty degeneration is also represented.

Besides these there are illustrated many of the anomalies, such as supernumerary teeth, union of two teeth, either through the medium of the cementum or by germination; irregularities in the position of the teeth and their repair after fracture by union. The remaining numbers are very nearly ready.

In a private letter Prof. Wedl states he is preparing an extensive text book to accompany the Atlas.

This work promises to the profession a text book devoted especially to Dental pathology, and should be in the possession of every Dentist. T. B. H.

